# Adverse effects of chronic exposure to nonylphenol on non-alcoholic fatty liver disease in male rats

**DOI:** 10.1371/journal.pone.0180218

**Published:** 2017-07-07

**Authors:** Jie Yu, Xuesong Yang, Ya Luo, Xuefeng Yang, Mengxue Yang, Jin Yang, Jie Zhou, Feng Gao, Liting He, Jie Xu

**Affiliations:** 1 School of Public Health, Zunyi Medical University, Zunyi, Guizhou, P.R. of China; 2 Department of Gastrointestinal Surgery, Affiliated Hospital of Zunyi Medical University, Zunyi, Guizhou, P.R. China; 3 Department of Endocrinology, The First Affiliated Hospital of Zunyi Medical University, Zunyi, Guizhou, P.R. China; East Tennessee State University, UNITED STATES

## Abstract

Endocrine-disrupting chemical (EDC) has been thought to play a role in non-alcoholic fatty liver disease (NAFLD). However, the toxic effects of Nonylphenol (NP), an EDC, on non-alcoholic fatty liver disease have never been elaborated. This study aimed to investigate whether exposure to NP could induce NAFDL, a promoting effect of high-sucrose-high-fat diet (HSHFD) on the adverse effects caused by NP was evaluated. Fourth eight male rats were assigned to four groups and each group was treated with a specific testing sample: normal-diet (ND) control group (C-ND); normal diet plus NP (180mg/kg/day) group (NP-ND); high-sucrose-high-fat-diet control group (C-HSHFD); HSHFD plus NP (180mg/kg/day) group (NP-HSHFD). At the age of 80 day, sonogram presents diffusely increased hepatic echogenicity in the NP-HSHFD group. The oblique diameter of liver in the NP-HSHFD group was significantly bigger than that in both the C-ND and NP-ND groups. At the age of 90 day, exposure to NP-HSHFD and NP-ND caused a significant increase in NP concentration in liver as compared to the C-ND group. The rats in the groups treated with NP+ND, HSHFD and NP+HSHFD produced significant increases in the body weight, fat weight and FMI, respectively, when compared to the C-ND group. The liver weight and hepatosomatic indexes (HIS) of rats in the NP-HSHFD group are higher than those in the C-HSHFD group. Exposure to NP-HSHFD induced the increases in plasma alanine aminotransferase (ALT), aspartate aminotransferase (AST), cholesterol (TC), triglyceride (TG) and low density lipoprotein (LDL) as compared to the C-ND group. Morphological examination of liver tissue from rats exposed to NP+HSHFD shown steatosis with marked accumulation of lipid droplets, hepatocellular ballooning degeneration and inflammatory cell infiltration. Chronic exposure to NP might induce NAFLD in male rats. The high-sucrose-high-fat diet accelerates and exacerbates the development of NAFLD caused by NP exposure.

## Introduction

Non-alcoholic fatty liver disease (NALFD) is an emerging public health problem that is an increasing cause of cirrhosis and hepatocellular carcinoma [1]. Approximately one billion people worldwide are affected by NAFLD [2], a chronic metabolic disorder caused by an excess of lipid storage within the liver (referred to as steatosis), of which obesity and type 2 diabetes are frequent comorbidities [3]. In addition to lifestyle factors, environmental chemicals acting as endocrine disruptors have been thought to play a role in NALFD.

Nonylphenol (NP), a high-production industrial chemical and component of polycarbonate plastics, is ubiquitous in the environment [4, 5]. In China, production of NP polyethoxylates (NPnEO) is about 50,000 ton per year, and approximately 70% of NP is used for the production of synthetic detergents [6]. NP is considered an endocrine-disrupting chemical (EDC), with estrogenic and thyroid hormone effects observed in experimental and epidemiological studies [7, 8]. Such wide application and use makes NP detectable in urine samples worldwide [9]. NP accumulation in human adipose tissue has also been observed [10].

Evidence is emerging regarding the underlying pathophysiology of NP and metabolic dysregulation. Exposure to NP elevates reactive oxygen species and induces oxidative stress in various tissues, including the liver [11]. Jubendradass (2011) demonstrated that short-term exposure to NP induces hypoglycemia, hyperinsulinemia, and oxidative stress in the pancreas of adult rats [12]. In another study, Hao (2012) revealed that NP promotes adipocyte differentiation and induces obesity in mice [13]. However, whether exposure to NP ultimately leads to NAFDL has not yet been studied. Therefore, in the present study, we sought to investigate whether exposure to NP causes NAFDL. Furthermore, any promoting effect of a high-sucrose/high-fat diet on the toxic effects induced by NP was also evaluated.

## Material and method

### Ethics statement

All procedures were conducted at Zunyi Medical University and were performed in strict accordance with the guidelines and regulations set forth by the Zunyi Medical University ethics committee with full approval from its Animal Care and Use Committee.

### Reagents

The NP was purchased from the Tokyo Chemical Co., Ltd (Tokyo, Japan). Automatic biochemical analyzer was purchased from Beckman Coulter (Villepinte, France). Low-speed centrifuge was purchased from Eppendorf (Schönenbuch, Switzerland). High performance liquid chromatography was purchased from Waters Technologies (Milford, MA, USA). Doppler color ultrasound was purchased from Philips Ultrasound (CA, USA). All other chemicals were commercially available. All chemical purities were at least 99%.

### Animals and treatments

Fourth-eight male Sprague-Dawley (SD) rats from the Animal Center of the Third Military Medical University (Chongqing, China) were fed commercial rat chow for 4 weeks. The 48 rats were assigned into four groups (n = 12 per group), and each group was treated with a specific testing sample for 90 days. The 48 male rats were orally exposed to NP (180 mg/kg/day) or to groundnut oil alone (vehicle control, 2 ml/kg/day). The first group was fed a normal diet (SCXK2012-0012, The Third Affiliated Hospital of Third Military Medical University) and received gavage with groundnut oil alone, and served as the normal-diet (ND) control (C-ND); the second group was fed a normal diet and received gavage with NP (180 mg/kg/day) (NP-ND); the third group was fed a high-sucrose/high-fat diet (HSHFD) (SCXK2012-0012, The Third Affiliated Hospital of Third Military Medical University), and served as the high-sucrose/high-fat-diet control (C-HSHFD); the fourth group was fed a HSHFD and received gavage with NP (180 mg/kg/d) (NP-HSHFD). The composition of the HSHFD included lard (10%), sugar (10%), and eggs (8%) in addition to a normal diet (70%). The animals were maintained under controlled temperature (20±1°C) and humidity (60±5%), on a 12-hr light (09:00–21:00hr), 12-hr dark (21:00–09:00 hr) cycle. Food and water were freely available. All procedures involving the use of laboratory animals were in accordance with Animal Care and Use Guidelines in China.

### High-performance liquid chromatography

Hepatic concentrations of NP were measured using the high-performance liquid chromatography (HPLC) technique described by Remane et al. (2016). Approximately 0.5 g minced samples of liver were spiked with hexane/diethyl ether (8/2, v/v) and extracted with 6 mL of 1 N HCl:acetonitrile (1:1) in two homogenization and centrifugation (2 × 1000×*g* for 15 min each time) steps. HPLC analysis was performed using a Waters 2695 HPLC system (Waters, Milford, MA, USA) equipped with a quaternary solvent delivery system, an auto-sampler with a 100-μl sample loop, a PDA detector, a column oven, and a data station running the Empower data software. A CAPCELL PAK C18 column (150 mm × 4.6 mm, 3 μm; Chuo-ku, Tokyo, Japan) was used for separation and was maintained at 25°C. The mobile phase was composed of water (eluent A) and acetonitrile (eluent B), and the elution was carried out in gradient mode at a flow rate of 0.7 ml/min. The gradient program was as follows: 0–2 min, 30% B; 2–6 min, 30–70% B; 6–15 min, 70% B; 15–16 min, 70–100% B; 16–25 min, 100% B. Finally, the initial condition was re-introduced over 0.01 min, and the equilibration time was 5 min. The injection volume was 10 μl. The detection wavelength was 242 nm for all the analytes except megestrol acetate and melengestrol acetate (288 nm) [14].

### Detection of oblique diameter of liver

The oblique diameter of liver in rat was measured by Doppler color ultrasound (US) at the age of 80 days. The measurements were made with the rats resting comfortably in the supine position. The oblique diameter of right liver lobe is the biggest vertical diameter when the rats were measured from the abdomen to back by Doppler color ultrasound.

### Detection of body weight, liver weight, fat weight, hepatosomatic indexes (HIS) and fat mass index (FMI)

At the age of 90 days, the rats were weighed. After weighing, pentobarbital sodium was given at a dose of 20 mg/kg intraperitoneal injection to induce anesthesia. Blood was collected from the abdominal aorta in heparinized vials after anaesthesia for biochemical studies. After the blood was collected, the rats were dead with cardiac arrests, the rats were then decapitated because we would collect brain tissue to detect couples of markers. The ethics committee of Zunyi Medical University reviewed and approved the decapitation method. Subsequently, rats were killed, livers, testis, and epididymis adipose were rapidly dissected, weighed, frozen in liquid nitrogen and stored at −80°C. The HIS and FMI were calculated by comparing the respective organ weights to the total body weight (organ weight/total body weight × 100). HIS = liver weight/total body weight × 100. FMI = testis and epididymis adipose weight/total body weight × 100.

### Biochemical studies

Plasma was separated by centrifuging the blood at 4,000 rpm at 4°C for 10 min and was stored below −80°C until analysis. Plasma alanine aminotransferase (ALT), aspartate aminotransferase (AST), total cholesterol (TC), triglyceride (TG), and low density lipoprotein (LDL) activities were assayed using the colorimetric method of Reitman and Frankel, as described by Bergmeyer [15].

### Liver biopsies

Liver specimens were taken immediately after the rats were sacrificed. Paraffin-embedded sections of liver were fixed in 10% formalin and stained with hematoxylin-eosin (HE) to detect hepatic steatosis, inflammation, and necrosis. Fatty change was graded according to the percentage of hepatocytes containing macrovesicular fat (grade 1: 0–25%; grade 2: 26–50%; grade 3: 51–75%; grade 4, 76–100%). The degree of inflammation and necrosis was expressed as the mean of 10 different fields on each slide; each field had been classified on a scale of 0–3 (0: normal; 1: mild; 2: moderate; 3: severe) [16].

### Statistical analysis

The statistical analyses were performed with SPSS software, version 13.0 for Windows (SPSS Inc., Chicago, IL). Values of all variables are presented as mean and standard deviation. One-way analysis of variance (ANOVA) with Tukey’s HSD as posthoc test and LSD-t test were used to determine the effects of different treatments. A P-value <0.05 was considered as the level of statistical significance.

## Results

### Liver Doppler ultrasound

#### Ultrasound imaging

**Fig 1:**
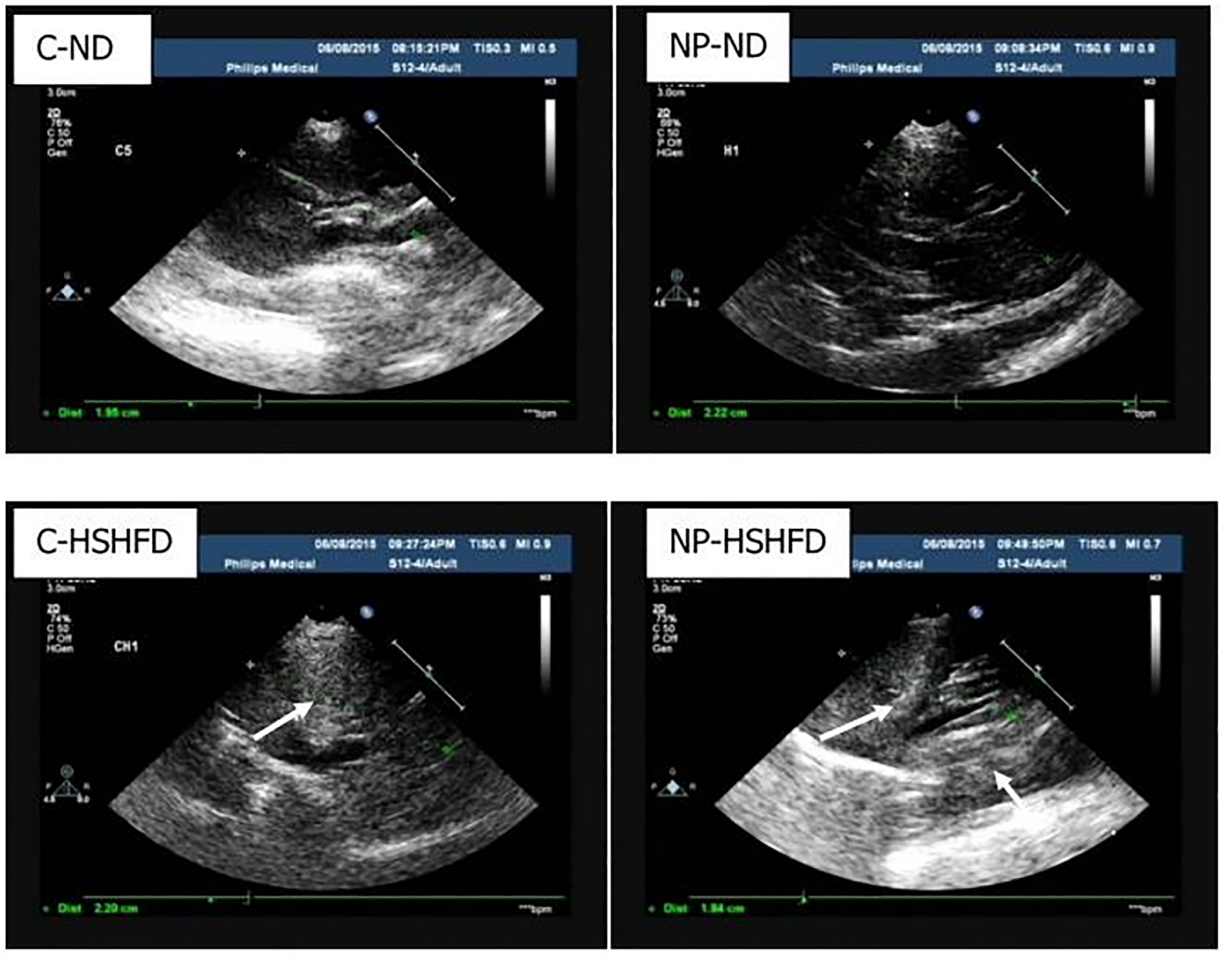
Liver ultrasound imaging. The hyperechogenicity of liver parenchyma (arrowheads). C-ND: normal-diet control; NP-ND: normal-diet plus NP; HSHFD: high-fat-diet control; NP- HSHFD: high fat diet plus NP.

All animals were examined by US imaging after 80 days of gavage. Alterations were represented by changes in liver echogenicity. In the C-ND group, a sonogram demonstrated homogeneous liver parenchyma, with medium-level echogenicity and a regular hepatic surface. The sonogram presented diffusely increased hepatic echogenicity in the NP-HSHFD group when compared with the C-ND group, implying that exposure to NP and HSHFD could lead to fatty liver (Fig 1).

### Oblique diameter of liver

**Fig 2:**
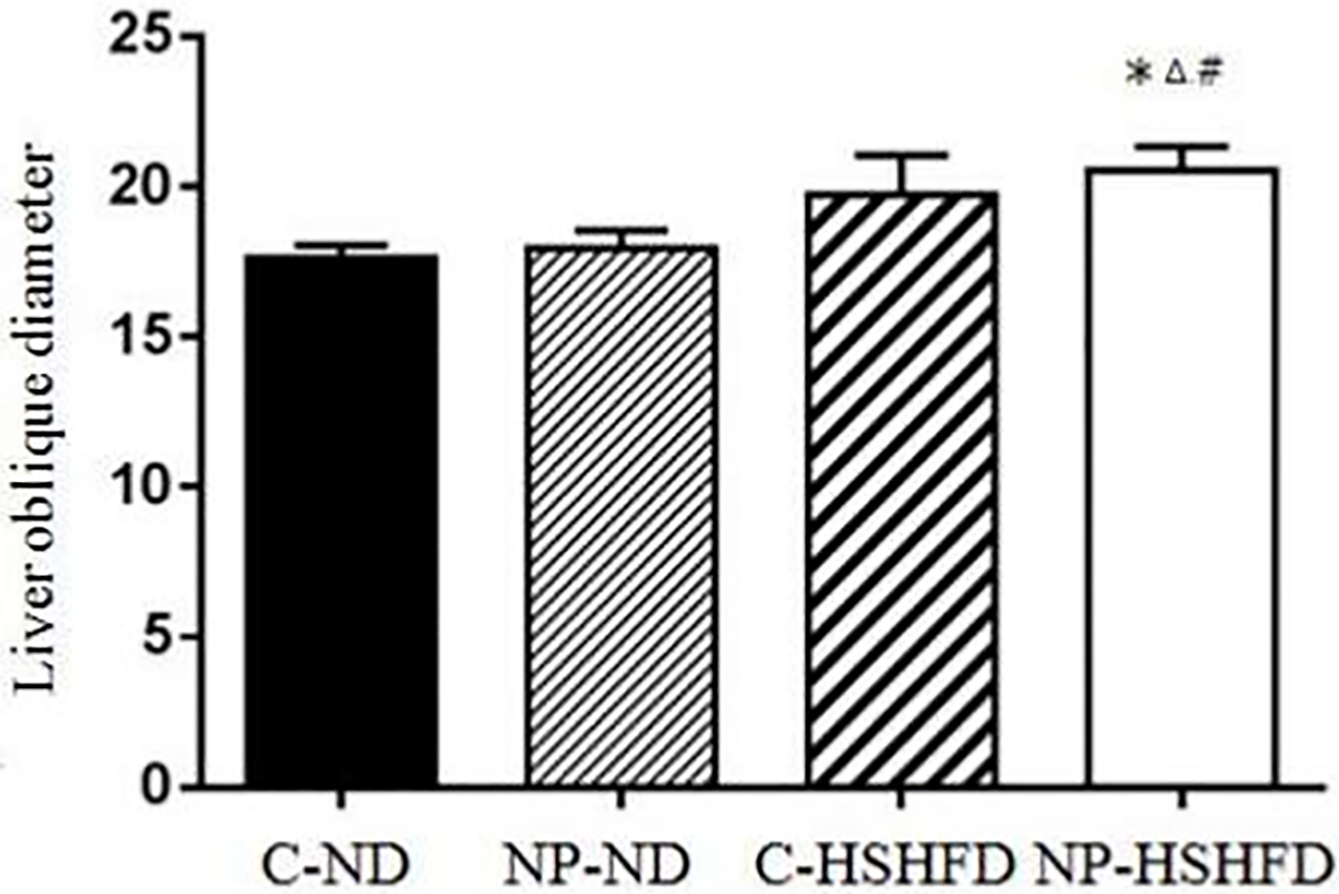
Comparison in the oblique diameter of liver in different treatment group. NP concentration in liver was ng/ml supernatant fraction of a liver homogenate. All values are expressed as mean ± standard deviation (n = 12) * NP-HSHFD vs control, *p*<0.05; ^#^ NP-HSHFD vs NP-ND, *p*<0.05; ^Δ^NP-HSHFD vs C-HSHFD, *p*<0.05.

The oblique diameter of the liver in the NP-HSHFD group was significantly larger than that in both the C-ND and NP-ND treatment groups (F = 13.671, *p*<0.05). The oblique diameter of the liver in the C-ND group was smaller than that in the NP-ND group. There was a significant difference between these two treatment groups. A significant difference in the oblique diameter of the liver compared with the C-HSHFD group was seen in the NP-HSHFD group (*p*<0.05; Fig 2).

### NP concentration in liver

**Fig 3:**
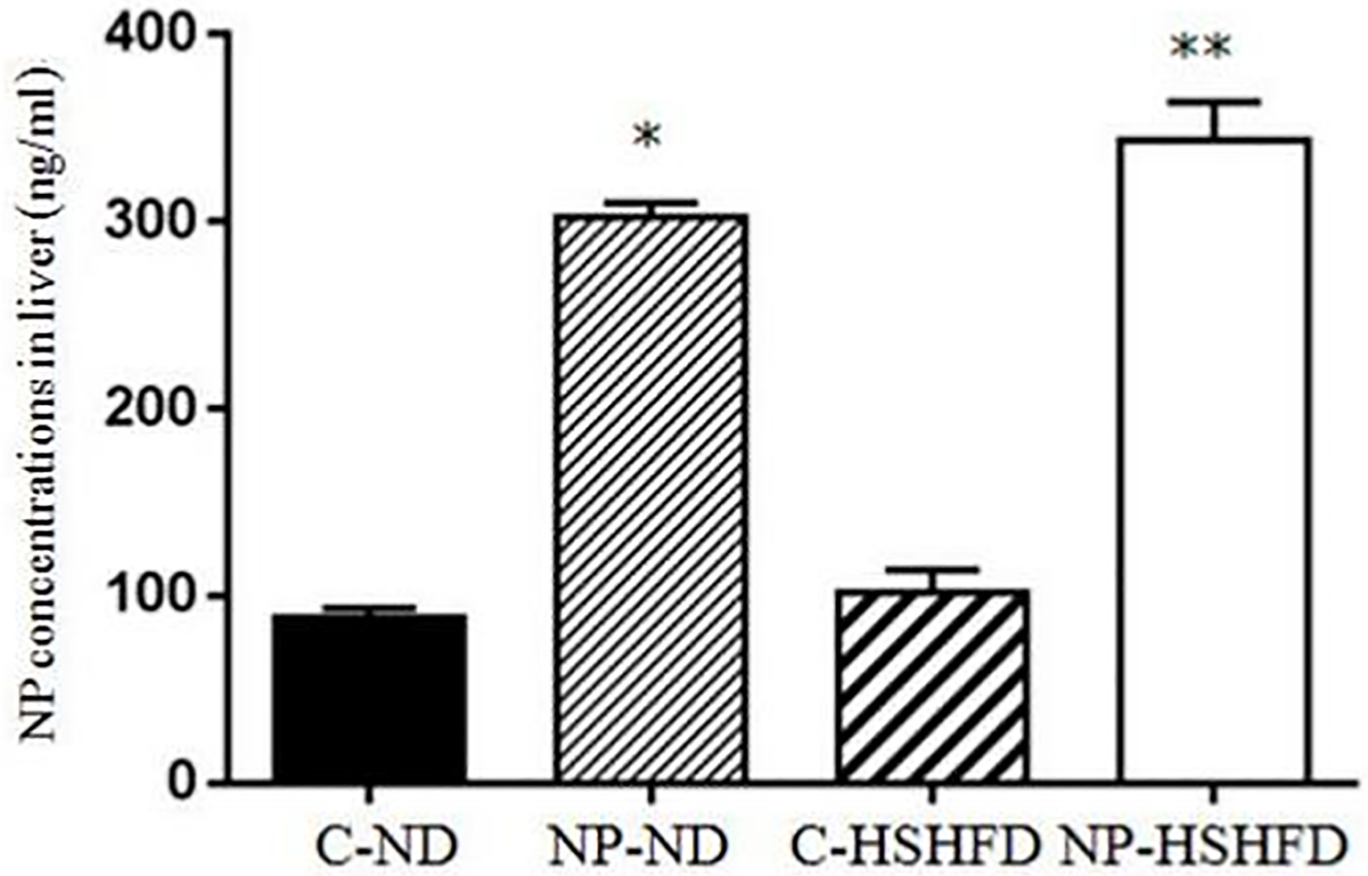
Comparison in NP concentrations in liver in treatment groups. NP concentration in liver was ng/ml supernatant fraction of a liver homogenate. All values are expressed as mean ± standard deviation (n = 12) * NP-ND vs control, *p*<0.05; ** NP-HSHFD vs control, *p*<0.05.

Exposure to NP-HSHFD and NP-ND caused a significant increase (F = 549.85, *p*<0.05) in NP concentration in the liver as compared with the concentration in the C-ND group. No differences compared with C-ND were seen in the C-HSHFD group (*p* >0.05; Fig 3).

### Body weight, liver weight, fat weight, HIS and FMI

**Fig 4:**
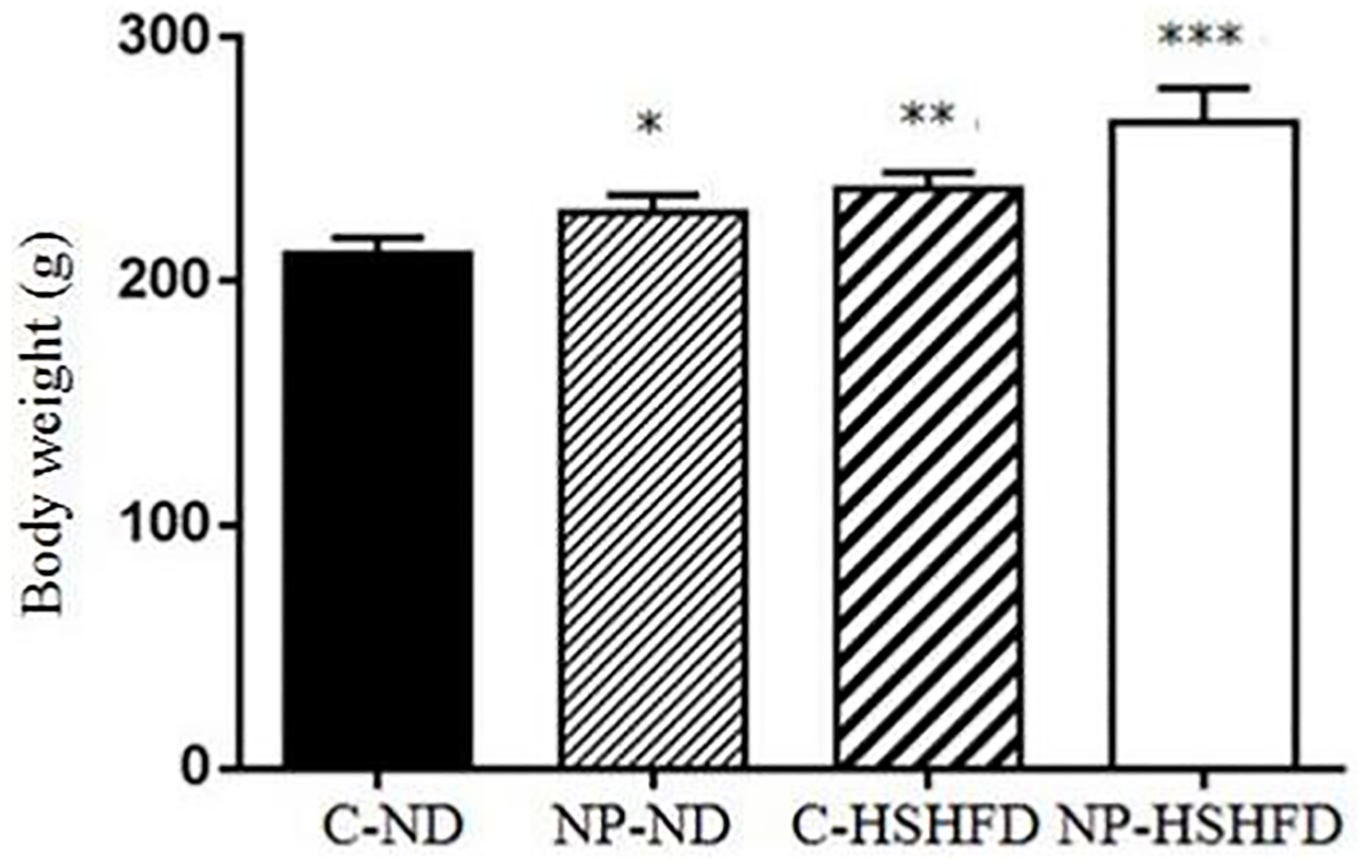
Comparison in body weight in different treatment groups. All values are expressed as mean ± standard deviation (n = 12) * NP-ND vs control, *p*<0.05; ** HSHFD vs control, *p*<0.05; *** NP-HSHFD vs control, *p*<0.05.

**Fig 5:**
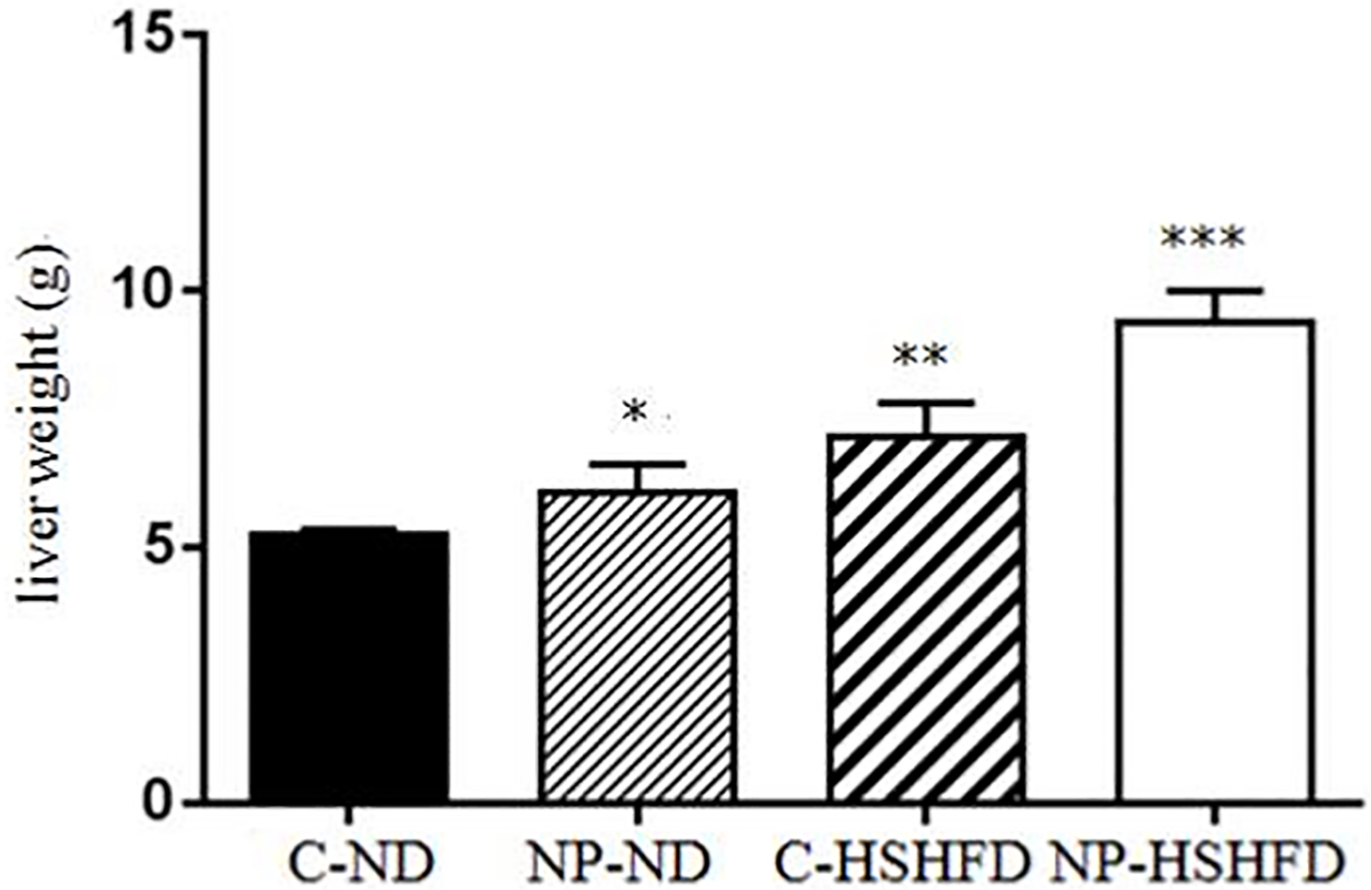
Comparison in liver weight in different treatment groups. All values are expressed as mean ± standard deviation (n = 12) * NP-ND vs control, *p*<0.05; ** HSHFD vs control, *p*<0.05; *** NP-HSHFD vs control, *p*<0.05.

**Fig 6:**
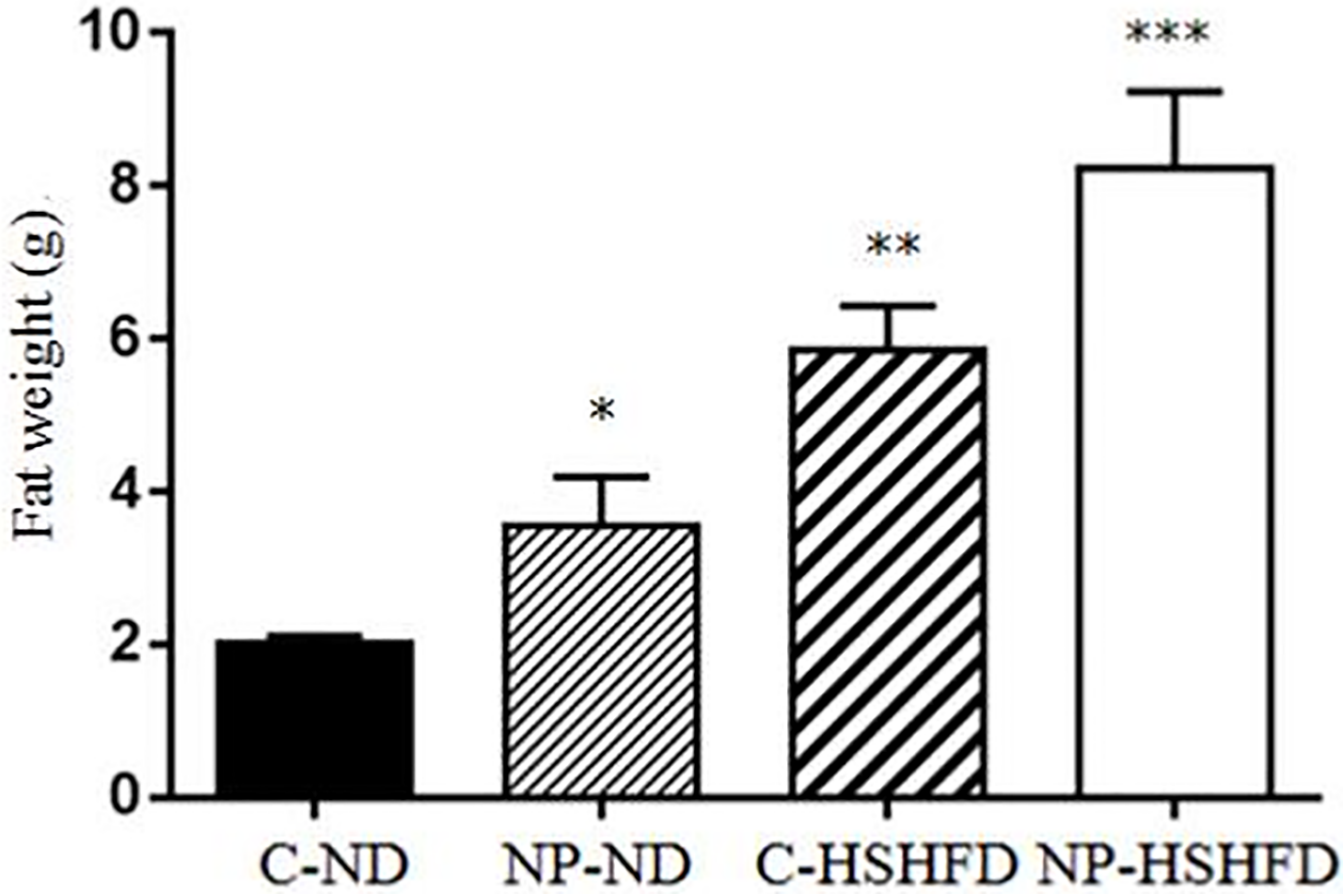
Comparison in fat weight in different treatment groups. All values are expressed as mean ± standard deviation (n = 12) * NP-ND vs control, *p*<0.05; ** HSHFD vs control, *p*<0.05; *** NP-HSHFD vs control, *p*<0.05.

**Fig 7:**
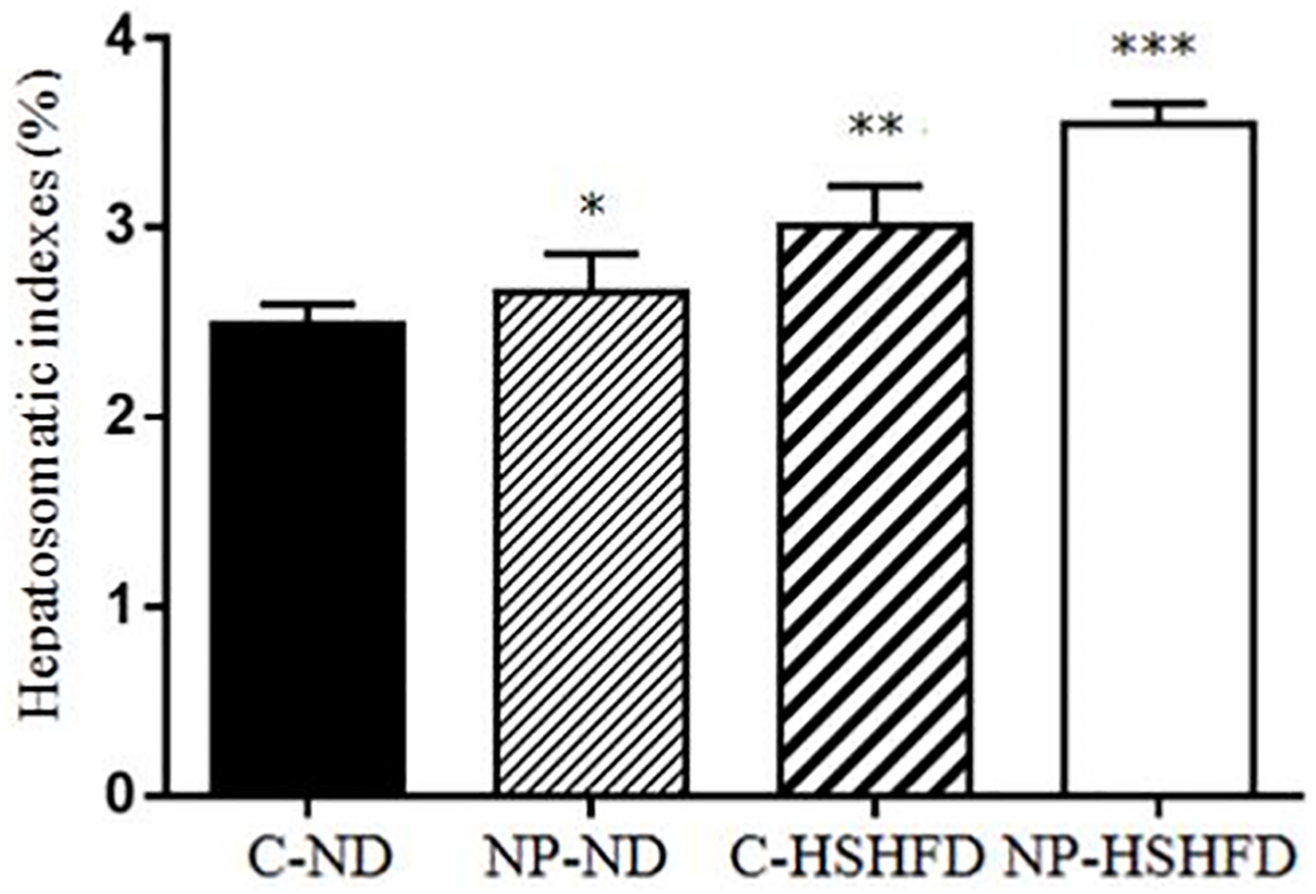
Comparison in hepatosomatic indexes in different treatment groups. All values are expressed as mean ± standard deviation (n = 12) *NP-ND vs control, *p*<0.05; **HSHFD vs control, *p*<0.05; *** NP-HSHFD vs control, *p*<0.05.

**Fig 8:**
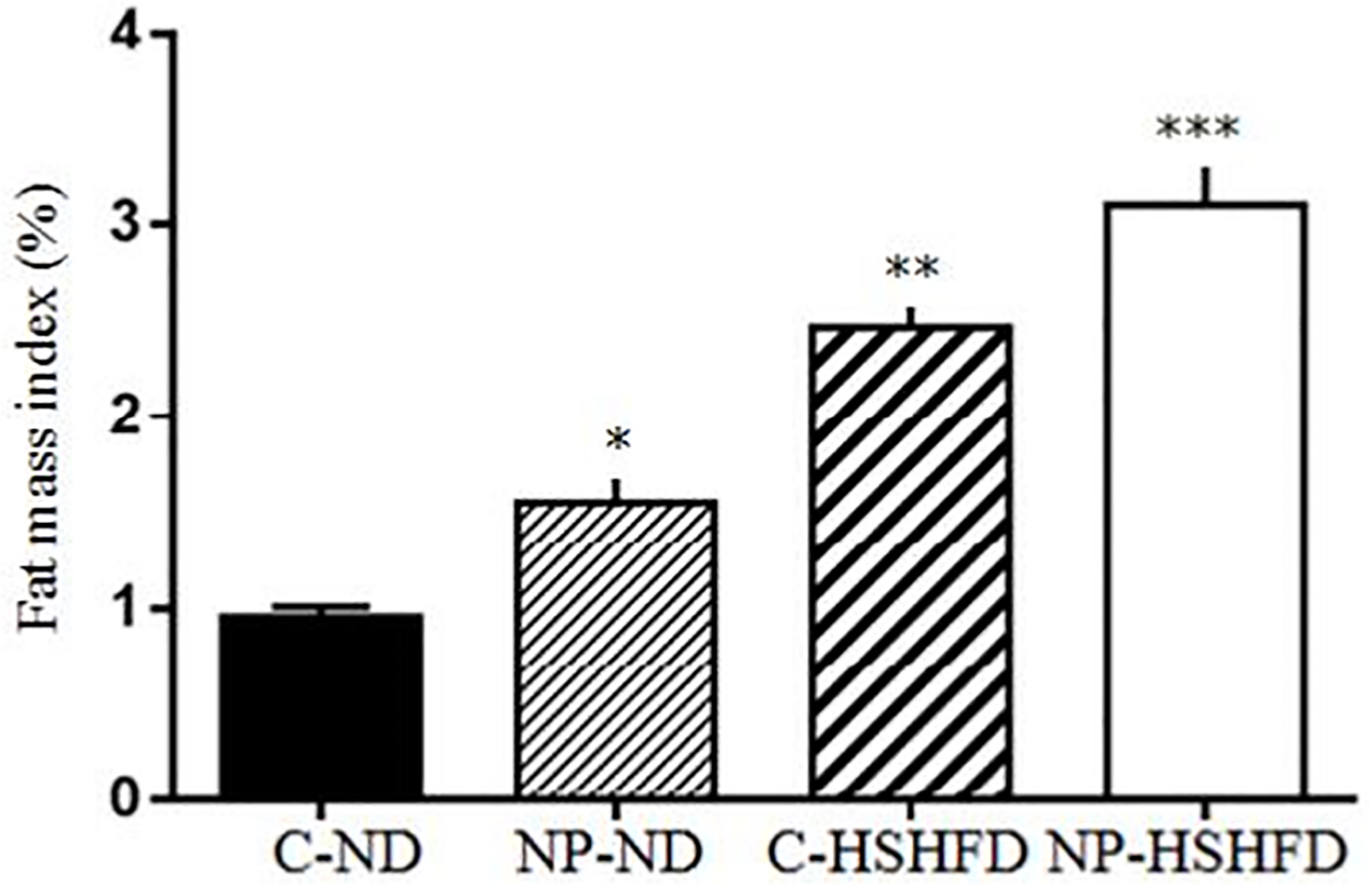
Comparison in fat mass index in different treatment groups. All values are expressed as mean ± standard deviation (n = 12) *NP-ND vs control, *p*<0.05; **HSHFD vs control, *p*<0.05; *** NP-HSHFD vs control, *p*<0.05.

The rats in the groups treated with NP-ND, HSHFD and NP-HSHFD showed significant increases in body weight (F = 29.56, *p*<0.05), fat weight (F = 83.85, *p*<0.05), and FMI (F = 91.08, *p*<0.05), compared with the C-ND group. The liver weight (F = 57.15, *p*<0.05) and HIS (F = 39.70, *p*<0.05) of rats in the NP-HSHFD group were higher than those in the C-HSHFD group (Figs 4–8).

### Serum biochemical indexes measurement

**Fig 9:**
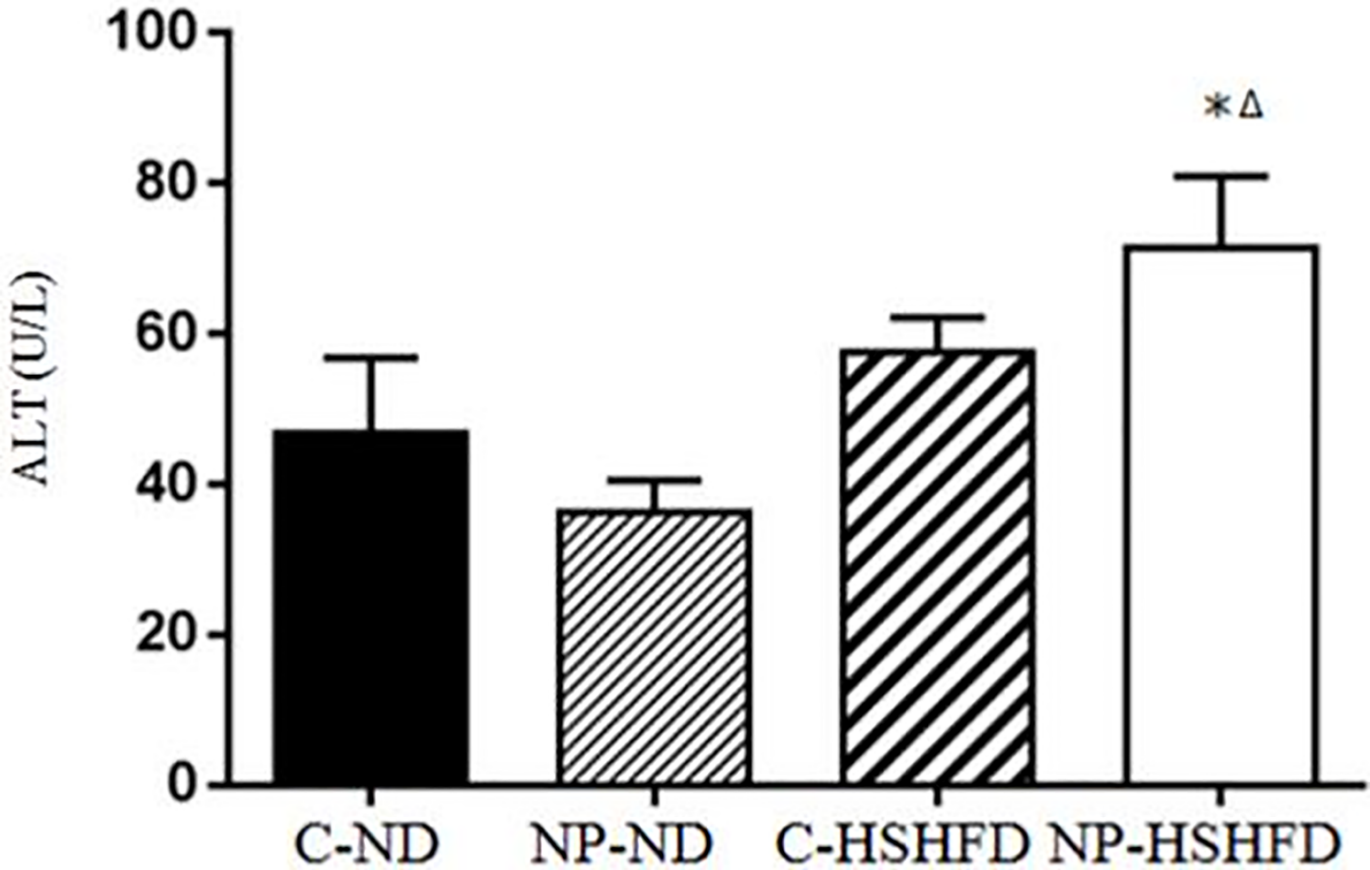
Comparison in alanine aminotransferase in different treatment groups. All values are expressed as mean ± standard deviation (n = 12) *NP-HSHFD vs control; ^Δ^NP-HSHFD vs C-HSHFD, *p*<0.05.

**Fig 10:**
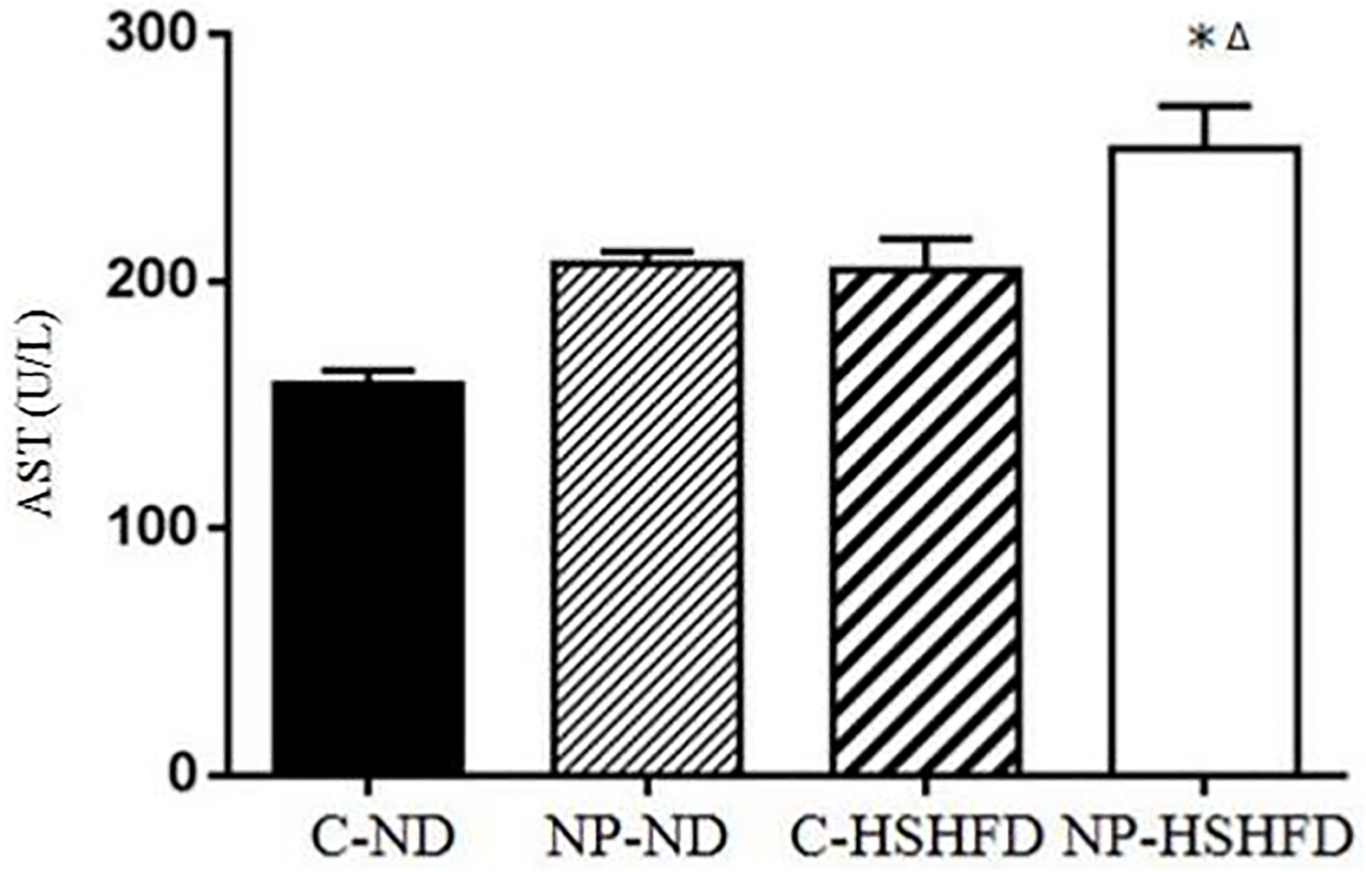
Comparison in aspartate aminotransferase in different treatment groups. All values are expressed as mean ± standard deviation (n = 12) *NP-HSHFD vs control; ^Δ^NP-HSHFD vs C-HSHFD, *p*<0.05.

**Fig 11:**
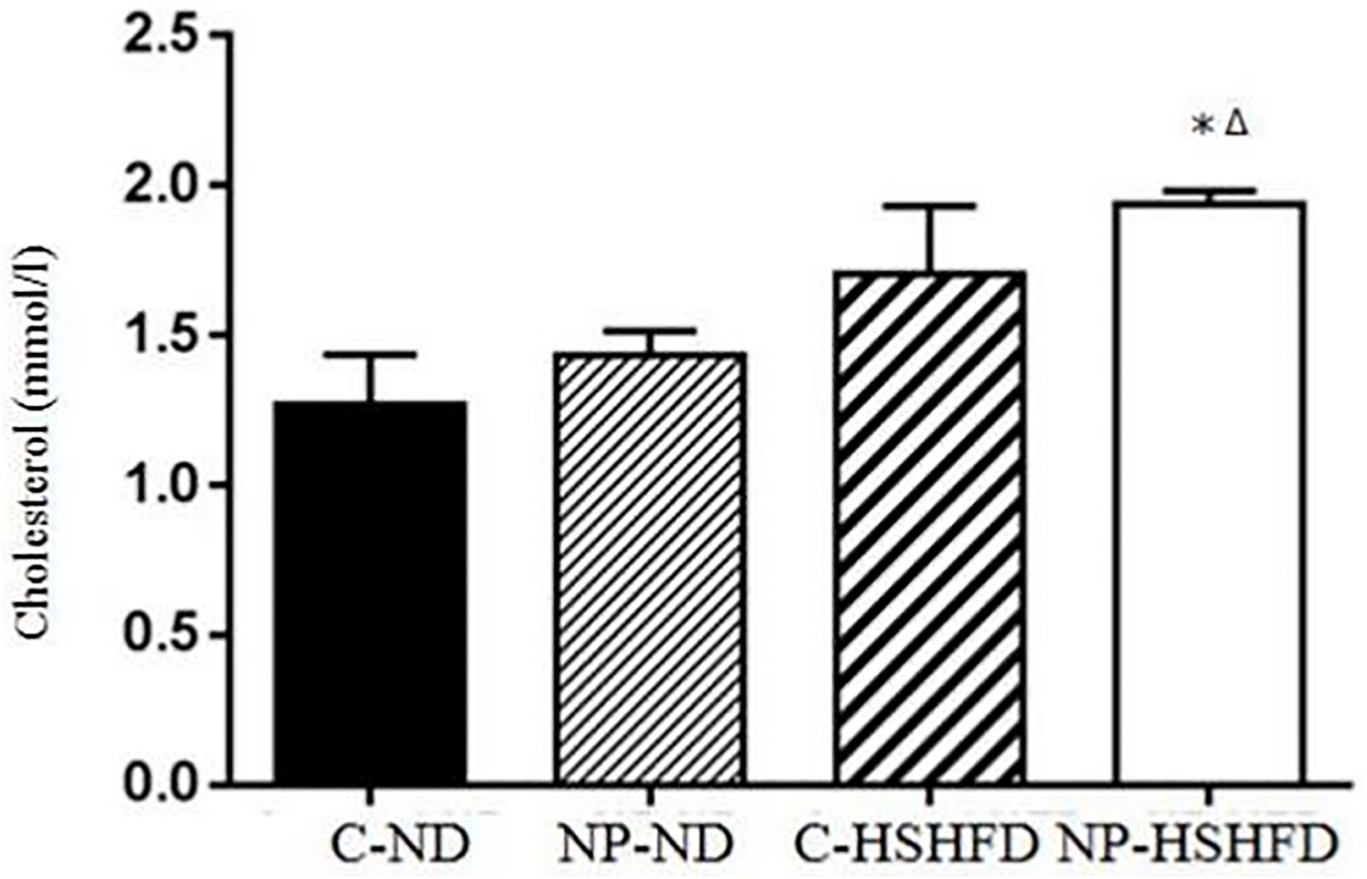
Comparison in cholesterol in different treatment groups. All values are expressed as mean ± standard deviation (n = 12) *NP-HSHFD vs control; ^Δ^NP-HSHFD vs C-HSHFD, *p*<0.05.

**Fig 12:**
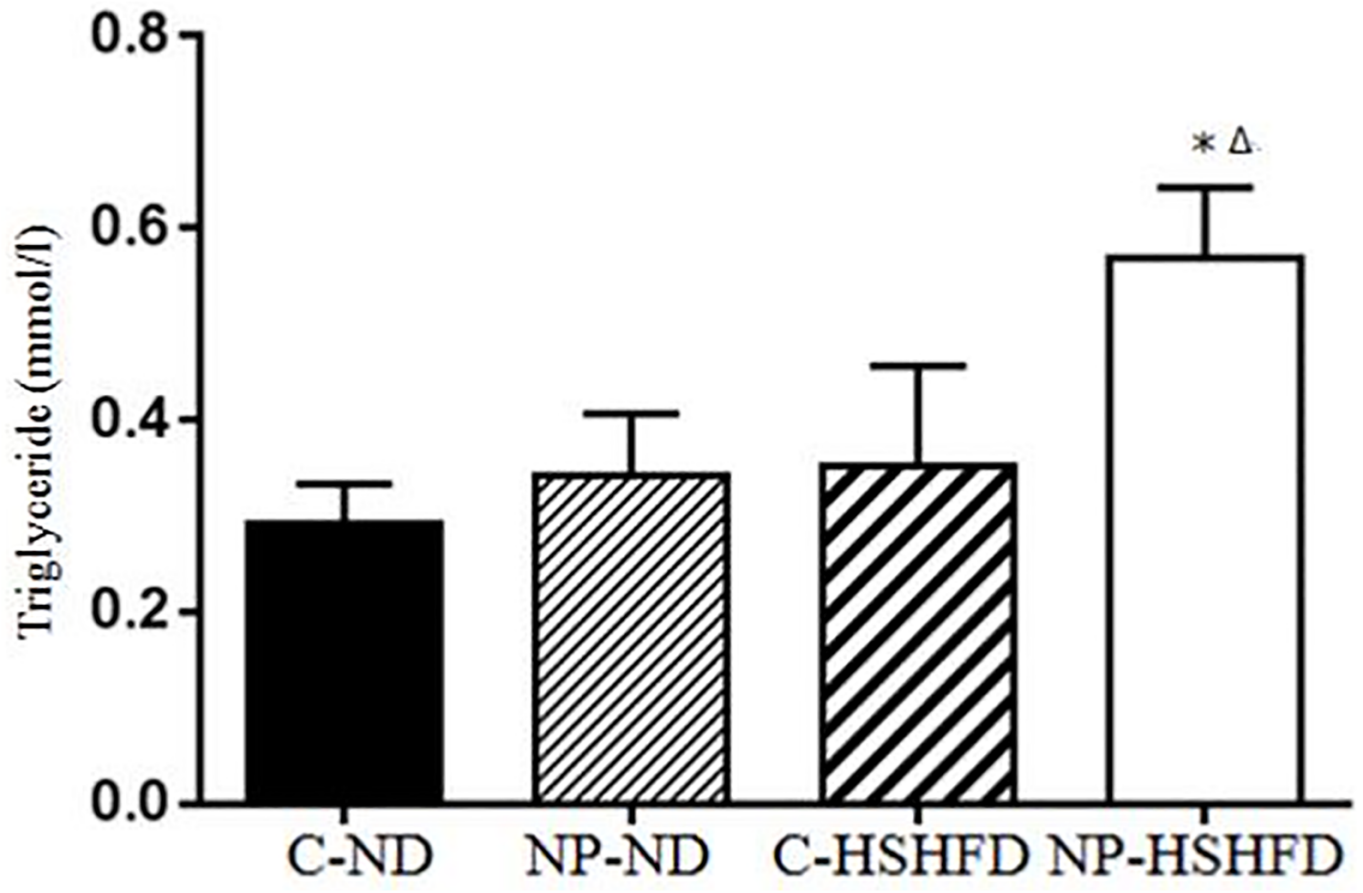
Comparison in triglyceride in different treatment groups. All values are expressed as mean ± standard deviation (n = 12) *NP-HSHFD vs control; ^Δ^NP-HSHFD vs C-HSHFD, *p*<0.05.

**Fig 13:**
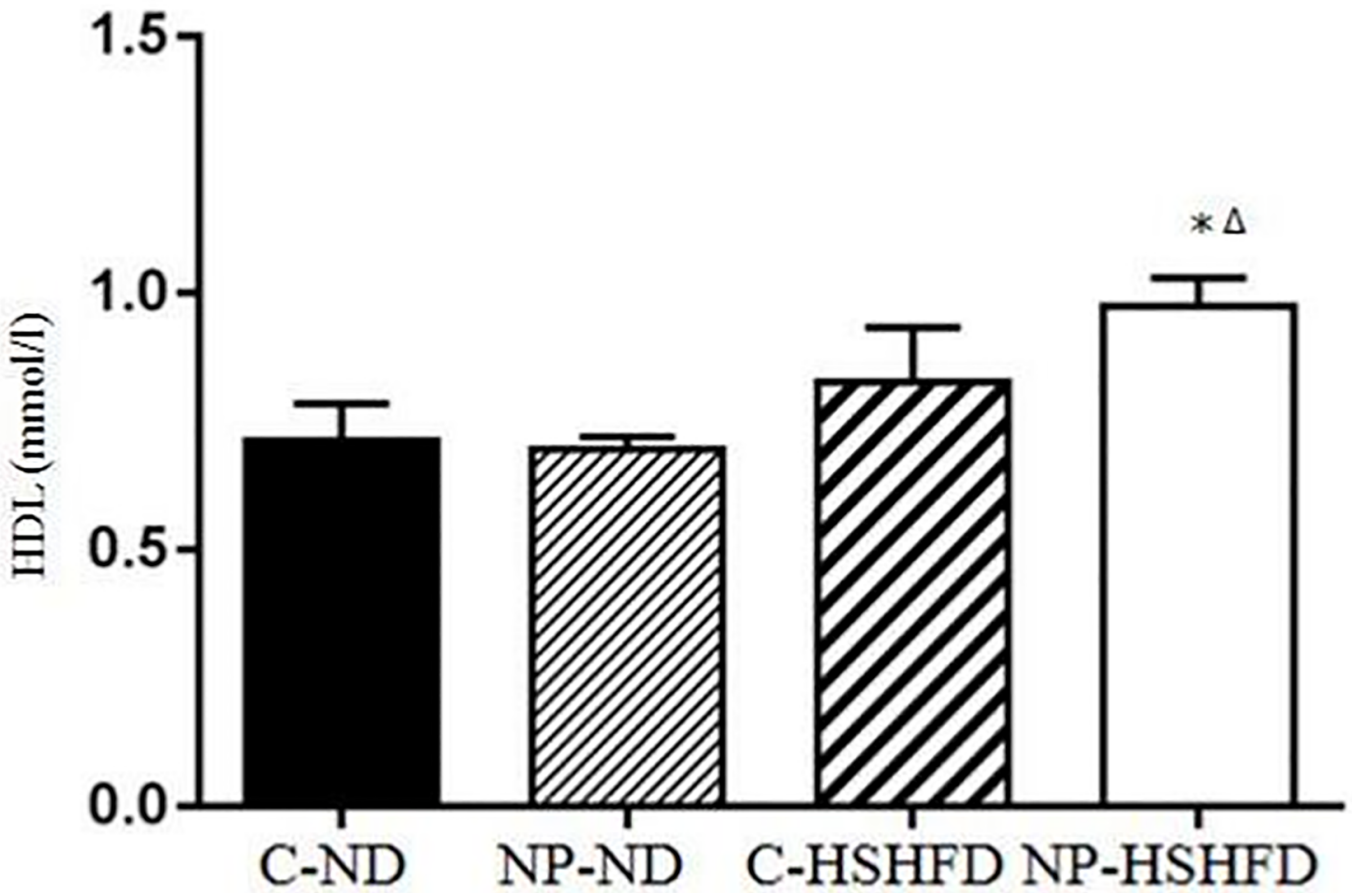
Comparison in high density lipoprotein in different treatment groups. All values are expressed as mean ± standard deviation (n = 12) *NP-HSHFD vs control; ^Δ^NP-HSHFD vs C-HSHFD, *p*<0.05.

**Fig 14:**
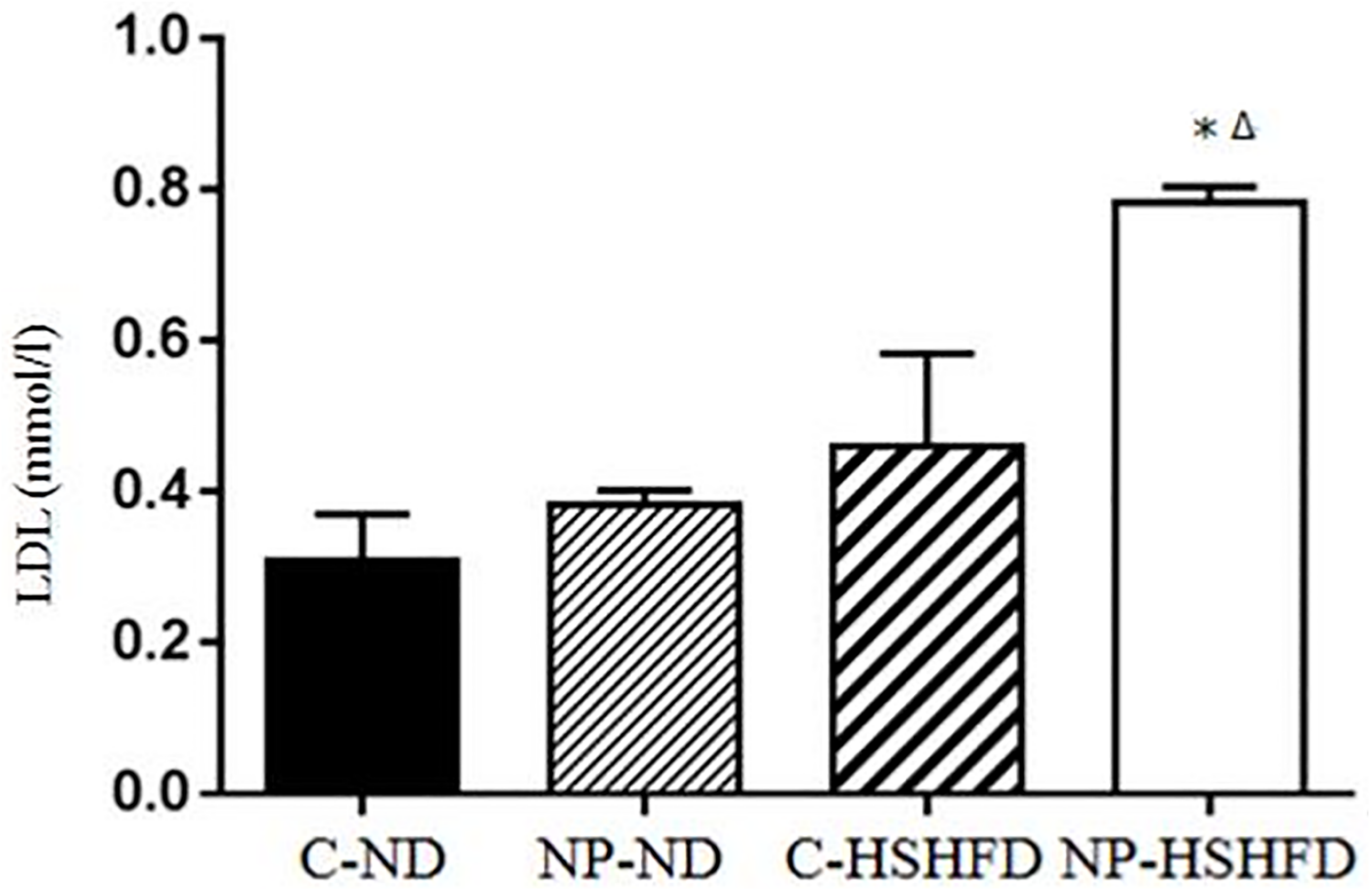
Comparison in low density lipoprotein in different treatment groups. All values are expressed as mean ± standard deviation (n = 12) * NP-HSHFD vs control; ^Δ^NP-HSHFD vs C-HSHFD, *p*<0.05.

Exposure to NP-HSHFD induced increases in plasma ALT (*F* = 8.74, *p*<0.05), AST (*F* = 7.42, *p*<0.05), TC (*F* = 7.08, *p*<0.05), TG (*F* = 4.72, *p*<0.05), HDL (*F* = 7.35, *p*<0.05), and LDL (*F* = 3.50, *p*<0.05) compared with the C-ND and C-HSHFD groups (Figs 9–14).

### Morphological changes in liver tissue

There were no significant morphological changes in the HE-stained hepatic cells in the treatment groups fed a normal diet. Further, there was no significant degeneration or infiltration of inflammatory cells, nor evidence of liposomal or inflammatory cell infiltration.

**Fig 15:**
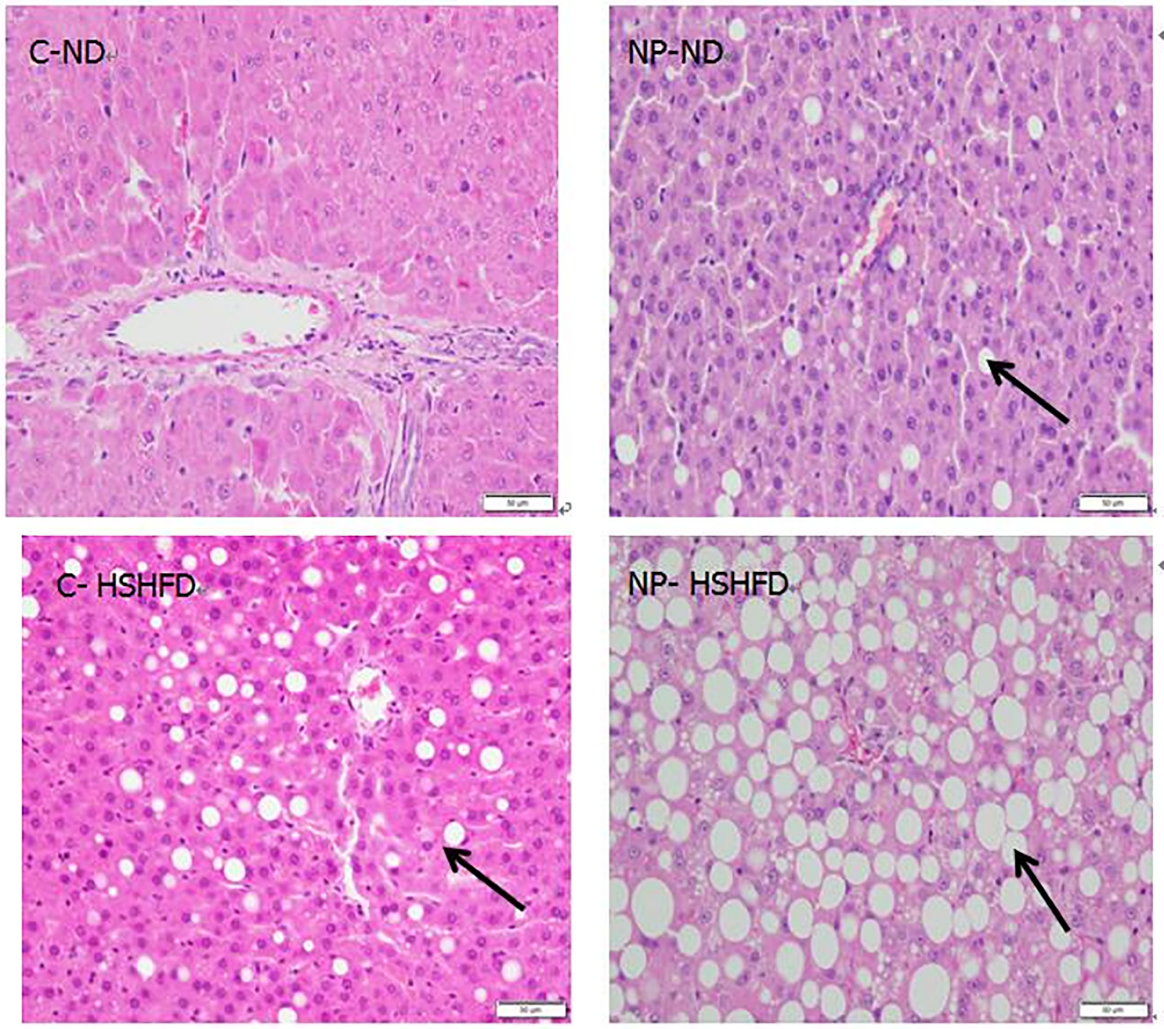
Morphological changes of the liver tissue. Lipid droplets in liver cells (Arrowheads) at the NP dose of 180mg/kg/day.

Morphological examination of HE-stained tissue showed steatosis with marked accumulation of lipid droplets in the NP-HSHFD group. In addition, there was ballooning degeneration of liver cells, and the steatosis appeared more severe, with inflammatory cell infiltration of the hepatic lobule and portal area (Fig 15).

## Discussion

To the best of our knowledge, there has been no previous report on the effects of chronic exposure to NP in terms of NAFLD. In the present study, we performed a series of toxicological test methods together with liver morphological observations to evaluate an association of NP with NAFLD. We observed that chronic exposure to NP might induce NAFLD in male rats; the HSHFD accelerates and exacerbates the development of NAFLD caused by NP exposure.

NPs have the potential to interfere with the endocrine [17], immune [8], and nervous [5] systems. Previous studies have shown that exposure to EDC (e.g., bisphenol A, phthalates) resulted in multiple features of metabolic syndrome in rats, including hyperlipidemia and lipid accumulation [18, 19]. However, the toxic effect of NP on NAFLD has not yet been elaborated. To mimic the most common route of exposure to NP in animals, we used an oral route for NP administration and continued NP exposure for 90 days. In addition, consumption of a high-energy diet is prevalent throughout the world, so we were interested in understanding if NP exposure would increase susceptibility to NAFLD in rats weaned onto a HSHFD. As expected, we found that the HSHFD worsened the hepatic damage caused by NP in rats. Finally, NAFLD is more common in men than in women [20]. Because we have previously shown that exposure to NP, an estrogen-like EDC, leads to alterations in the balance of sex hormone metabolism in male rats, only male rats were chosen in this study.

In the present study we demonstrate that exposure to NP over approximately 3 months in male rats results in metabolic disorders, including increased NP concentration in the liver, increased liver mass, increased adipose tissue mass, liver dysfunction, and blood lipid abnormalities. These results clearly show the toxic effects of NP on liver function in rats. Blood enzymes such as ALT, AST are involved in the metabolism of amino acids. An increase in the levels of ALT and AST has been shown to correspond with liver damage [21], while increased TC, TG, and LDL levels may be indicative of liver damage [22, 23]. In the present study, we elucidated the toxicological effects of NP on selected biochemical endpoints in rat models. Our results indicate that chronic exposure to NP plus HSHFD led to increases in ALT, AST, TC, TG, HDL and LDL in the liver of rats, as compared with the C-ND group.

These findings confirm many of the observations made by Bhattacharya et al. and Jubendradass et al. Jubendradass et al. observed that administration of NP by oral gavage every day for 45 days induced increased levels of AST and ALT in the liver of rats [12]. The Bhattacharya research results indicated that levels of AST and ALT in the liver were stimulated to rise under NP treatment compared with controls. This study suggests that NP can alter the structures and biochemical parameters of the liver of animals [22]. Altered transaminase levels could probably be related to toxic injury induced by NP, which may stimulate liver repair through protein turnover. Thus, it is possible to say that NP causes damage in the liver of male rats.

The calculated HIS was found to be significantly greater in the NP-HSHFD group. HIS is known to be a potential biomarker to assess the toxic impact of environmental stressors. HIS plays a key role in carbohydrate metabolism in animals [24]. Hence, our observation that NP exposure could be a potential chemical stressor to disrupt glucose metabolism.

Morphological examination of tissues from rats exposed to NP plus HSHFD shows steatosis with marked accumulation of lipid droplets, hepatocellular ballooning degeneration, and inflammatory cell infiltration. These results are consistent with previous studies that have demonstrated extensive accumulation of lipids, large lipid droplets, profound ballooning degeneration, and increased inflammation in the liver of male rat offspring perinatally exposed to other environmental EDCs (bisphenol A), and fed on a high-fat diet [25]. The incidence of NAFLD has increased in parallel with increasing trends in overweight and obesity in the last 3 decades [26]. The cornerstones of NAFLD development may be in relation to modern lifestyle and a lack of exercise as well as a HSHFD. The mechanisms by which NP influences hepatic injury are not well understood. It has been postulated that NP may induce oxidative damage in the liver [11]. The morphological examination of liver fibrosis was not conducted in this study, hence, our study is a qualitative rather than a quantitative study.

The LD50 for NP was 1620 mg/KG (NP standard specification. Product ID: C15630000, Lot:20801. Germany). In addition, the dose of the 10–25 percent of LD50 for toxicant is used as the dose of subchronic exposure [27], therefore, in this study we designed an exposure dose for NP of 180mg/kg, which was merely a toxicological dose.

## Conclusion

Chronic exposure to NP might induce NAFLD in male rats, as indicated by increased NP concentration in the liver, increased liver mass, increased adipose tissue mass, liver dysfunction and blood lipid abnormality, together with morphological changes in the steatosis with marked accumulation of lipid droplets, hepatocellular ballooning degeneration, and inflammatory cell infiltration. These alterations imply that a HSHFD may accelerate and exacerbate the development of NAFLD caused by NP exposure. Exposure to an unhealthy diet along with exposure to EDCs (including NP) may be a risk factor for the development of NAFLD.

## Supporting information

S1 File Original data for the analysis of the adverse effects of chronic exposure to nonylphenol on non-alcoholic fatty liver disease in male rats. (SAV)
